# Long-term treatment of aged Long Evans rats with a dietary supplement containing neuroprotective peptides (N-PEP-12) to prevent brain aging: effects of three months daily treatment by oral gavage

**Published:** 2015

**Authors:** B Hutter-Paier, B Reininger-Gutmann, R Wronski, E Doppler, H Moessler

**Affiliations:** * QPS Austria, Grambach, Austria; ** Medizinische Universität Graz, Graz, Austria; *** EVER Neuro Pharma GmbH, Unterach, Austria

**Keywords:** N-PEP-12, brain aging, oral gavage, hippocampus

## Abstract

Aging is associated with morphological and functional changes in the brain, resulting in the deterioration of cognitive performance. Growth factors like BDNF are suggested to be involved in the regulation of age-related processes in the brain. A novel dietary supplement produced from purified nerve cell proteins, N-PEP-12, has shown to share properties with naturally occurring peptide growth factors by stimulating neurite outgrowth and beneficial effects on neuronal survival and protection against metabolic stress in cell cultures. The current study investigates the effects of long-term intake on age-dependent memory decline by assessing cognitive performance and synaptic density. All the experiments were performed in aged Long Evans rats randomly assigned to saline or N-PEP-12 once daily by gavage over a period of three months. Behavioral tests were performed in the Morris Water Maze after one, two and three months of treatment. Histological examinations were performed in the hippocampal formation and in the entorhinal cortex by measuring the synaptic density. This study shows that the oral intake of N-PEP-12 has beneficial effects on the cognitive performance of aged animals and that these effects go along with an increase in the synaptic density. Thus, N-PEP-12 may help maintain memory and learning performance during the aging process.

## Introduction

Aging is regarded as a multifactorial and complex process affecting all body systems including the brain. A decrease in the capacity of the working memory, slower information processing and decreased attention and concentration abilities are key characteristics. Approximately 86 billion neurons and estimated; 100 to 1000 trillion synapses in the human brain allow for compensation of deficits [**1**,**2**], however, the hippocampus and the dorsolateral prefrontal cortex are most vulnerable to aging [**3**] and are both involved in the cognitive processes. Recent studies have shown that age-related cognitive impairment was not associated with a loss of cortical neurons but to synaptic alterations in the hippocampus and prefrontal cortex [**3**]. Due to the different cognitive tasks of these brain areas, they showed different synaptic vulnerabilities to aging, characterized by a loss of silent synapses in the prefrontal cortex and by a loss of established synapses in the hippocampus. Morphological changes in the hippocampus are characterized by reductions of dendritic branching and of spine density-mainly in the CA1 region, changes in the innervation pattern of the hippocampus and by the rate of neurogenesis [**4**]. These changes are paralleled by behavioral and functional deficits in hippocampus-dependent learning and memory tasks.

Studies have shown that associative learning [**5**], long-term potentiation [**6**] and estrogen treatment [**7**] had a beneficial effect on the synaptic complexity. It is considered unlikely that a single factor or a single class of effector molecules is responsible for the age-related morphological changes in the hippocampus. However, brain-derived neurotrophic factor (BDNF) is suggested to be involved in the regulation of age-related processes in the hippocampus. In line, there is evidence that disturbances in the BDNF-system might result in hippocampal dysfunctions as seen in Alzheimer’s disease or major depression [**4**]. In this context, growth factors might play an important role in the maintenance of the hippocampal structure within the adult brain as they have a direct influence on the branching pattern and/ or generation, growth and plasticity of dendritic spines of hippocampal neurons. As they influence the transmitter systems projecting to the hippocampal formation, they affect neuronal plasticity and hippocampal volume and their expression is altered during aging [**4**].

N-PEP-12 is a dietary supplement produced from purified nerve cell proteins under international pharmaceutical standards and contains amino acids and bioactive peptides. Previous studies have shown that the peptides of N-PEP-12 share properties with naturally occurring peptide growth factors [**8**]. These experiments were performed in neuronal tissue cultures under detrimental conditions as observed during aging and have shown a stimulating effect on neurite outgrowth and beneficial effects on neuronal survival and protection against metabolic stress. 

Sophisticated manufacturing processes together with the chemical nature of these peptides prevent further enzymatic degradation in the gastrointestinal tract after oral intake. Gavage delivery studies with radiolabeled N-PEP-12 peptides in rats resulted in significant delivery to the central nervous system 40 to 60 minutes after delivery, with the highest concentrations found in the frontal cortex, hypothalamus and medulla oblongata (**Fig. 1**).

**Fig. 1:**
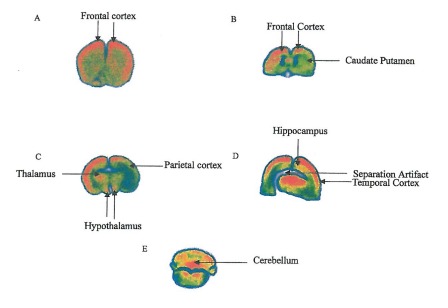
Brain distribution after gavage 125I N-PEP-12

The current study aims to show the effect of long-term intake of N-PEP-12 on age dependent memory decline in rats by assessing cognitive performance and the concomitant changes of synaptic density in the hippocampal formation.

## Materials and Methods

Treatment and behavioral tests

This research was carried out in accordance with the Austrian guidelines for the care and use of laboratory animals and was approved by the Austrian Ministry of Science (GZ 66.010/45-Pr/4/99).

All the experiments were performed in an 18±1 month-old male and female Long Evans rats randomly assigned to treatment with N-PEP-12 (N=14; 8f/ 6m) or physiological saline (control group; N=13; 8f/ 5m). Animals were treated once daily with 2.5ml/ kg bodyweight by gavage over a period of three months. 

Behavioral tests in the Morris Water Maze (MWM) were designed to obtain information on learning (acquisition), long- and short-term memory (retrieval) and procedural learning. The MWM paradigm consisted of a radial swimming pool in which an escape platform was hidden under the surface of the water. The MWM was performed on days 27-30, days 57-60 and days 87-90. Each animal performed four swimming trials on each trial day. Animals were randomly put into the pool on different starting positions and had to find the shortest way to the escape platform. This task required spatial navigation using extra maze landmarks, which were provided by different pictures attached to the wall of the experimental room. The circular swimming pool used for the MWM had a diameter of 170 centimeters and a height of 45 centimeters. The inner surface of the pool was painted black so that the submerged transparent Plexiglas platform (15 centimeters in diameter) could not be detected from outside. The platform was always located at the exactly same position of the pool in the southeast quadrant. Computer-aided video tracking was used to measure escape latency (elapsed time from introducing the animal in the water until the animal reached the platform or after 90 seconds) and traveled distance. 

Statistical analysis was performed by using the H-test according to Kruskal and Wallis or ANOVA in case of normally distributed values; the Scheffe test was used for post hoc analyses. 

Histological examinations

Immediately after finishing the behavioral experiments in the MWM, four to six rats of each group were sacrificed by an overdose of Nembutal. For proper histological preparation, transcardial perfusion with physiological saline and formalin was performed. For complete fixation, the brains were transferred into 10% formalin for 24h and later on imbedded into paraffin. Slices of 3 µm thickness (Bregma Level ~ -4.00 mm) were cut on the microtome and incubated with an antibody against the vesicular protein synaptophysin. The immunoreaction was visualized by using an enzyme reaction (avidin-biotin-complex-method, peroxidase conjugated secondary antibody; diaminobenzidine was used as substrate).

Due to the correlation of synaptic density and synaptophysin immunoreactivity, a light microscopic quantification of synaptic counts was performed by using an image analyzing system (LUCIA-Nikon Photo Systems, Austria). The number of synaptophysin immunoreactive presynaptic terminals and the complete area covered by them were measured in the hippocampus (CA1 stratum radiatum, CA2 stratum radiatum, CA3 stratum radiatum, CA3 stratum lucidum, dentate gyrus lateral blade and dentate gyrus medial blade) and in the entorhinal cortex (layers 2 and 3).

## Results

Behavioral tests in the MWM

Escape Latency 

After one month of application, the animals in both treatment groups improved to a similar extent in their escape latency (trial days 27-30). No significant difference between both experimental groups could be detected although N-PEP-12 treated rats acquired the task faster.

After two months of application (trial day 57), N-PEP-12 treated rats remembered the original platform position from the trial days 27-30 indicating a stabilization of long-term memory and most likely also an improved procedural learning; there was a statistically significant difference (p=0.0049) on trial day 57 between the N-PEP-12 group and the saline treated controls. As N-PEP-12 treated animals showed a very low escape latency already on trial day 57, further improvement was challenged by a ceiling effect due to limits in swimming speed. The control animals showed a continuous improvement from trial days 57 to 60 but they always performed worse compared to N-PEP-12 treated animals.

After three months of application, a ceiling effect was observed in both treatment groups (trial days 87-90). 

Long-term memory was assessed by comparing the escape latencies between the last trial on day 30 and the first trial on day 57. Animals treated with N-PEP-12 improved their performance by 9.9 (mean±21.6 SD) seconds, whereas control animals deteriorated by 1.3 (mean±29.1 SD) seconds. Similar results were observed one month later (13.0±26.8 seconds in the N-PEP-12 group vs. 1.4±20.0 in the control group) indicating an improved long-term memory in N-PEP-12 treated rats.

The effect on long-term memory was also assessed by comparing escape latencies of the naïve training situation on day 27 with re-trainings on days 57 and 87 whereas greater differences better reflected the long-term memory. Animals treated with N-PEP-12 decreased the escape latency by 52.3% (mean 24.7±2.5 SD) from trial day 27 to 57 compared to a decrease of 24.7% (mean 12.0±3.4 SD) in animals of the control group. The similar decrease in the escape latency from trial day 27 to 87 of 59.0% (mean 22.3±4.2 SD) in the N-PEP-12 group and 54.1% (mean 27.9±2.4 SD) in the control group indicated that the repetition of training finally led to success in all treatment groups. We have to consider that all experiments were performed in old, but otherwise healthy animals without any obvious neurological disturbances. Therefore, it was expected that prolonged training would continuously increase the cognitive performance in both treatment arms.

Data on escape latency are presented in the box plot of **Fig. 1**. 

**Fig. 1:**
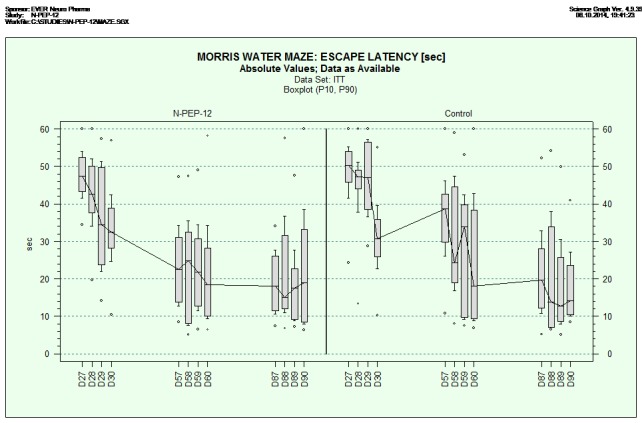
Behavioral tests in the Morris Water Maze. Box plot comparing the performance of rats receiving dietary supplement (N-PEP-12; n=14) or saline (control; n=13) over three months. The diagram shows the median escape latency with 10th and 90th percentile whiskers. Escape latency was significantly lower (p=0.0049) in the N-PEP-12 group when compared to saline on day 57

Traveled distance

Escape latency can severely be biased by the animal’s swimming speed. Thus, the length of the swimming path was calculated as an independent measure of learning and memory performance. The results reported from the N-PEP-12 treated and control animals were consistent with the effects observed on the escape latency.

After one month of application, the traveled distance of animals in both treatment groups improved to a similar extent (trial days 27-30). No significant difference between both experimental groups could be detected. After two months of application (trial day 57) there was a statistically significant difference (p=0.0133) between the N-PEP-12 group and the saline treated controls. The control animals showed a continuous improvement from trial days 57 to 60 but they always performed worse compared to N-PEP-12 treated animals. After three months of application, a ceiling effect was observed in both treatment groups (trial days 87-90). 

The assessment of long-term memory showed a reduced traveled distance in the N-PEP-12 group by 1.2 (mean±4.3 SD) meters from day 30 (last trial) until day 57 (first trial) and by 2.1 (mean±4.3) meters from day 60 (last trial) until day 87 (first trial). The travel distance of animals in the control group was of 1.3 (mean±4.5 SD) meters longer on day 57 (first trial) compared to day 30 (last trial) and 0.1 (mean±3.5 SD) meters longer on day 87 (first trial) compared to day 60 (last trial). 

These data on traveled distance were consistent with the observations in the escape latency, which indicated that the improved cognitive performance of animals treated with N-PEP-12 was not influenced by the motoric behavior of the animals.

Histological evaluation of synaptic density

After three months of treatment with N-PEP-12, the number of synaptophysin immunoreactive terminals increased to six out of seven investigated areas. Significant increases were observed in hippocampal areas “CA1 stratum radiatum”, “CA2 stratum radiatum”, “dentate gyrus medial blade”, “dentate gyrus lateral blade” and the entorhinal cortex. Similar results were after assessing the complete area covered by immunoreactive terminals.

**Fig. 2:**
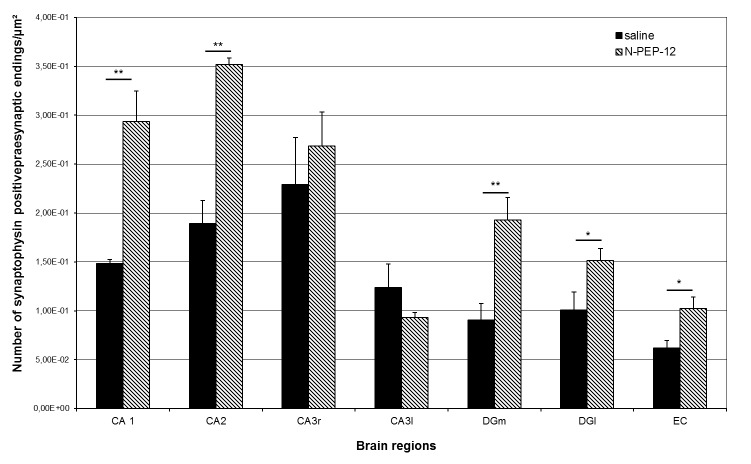
Histological evaluation of synaptic density. Number of synaptophysin positive presynaptic terminals in the saline (n=4) and N-PEP-12 (n=6) group shown as mean +SEM; *=p< 0.05, **=p< 0.001. CA1 = CA1 stratum radiatum, CA2 = CA2 stratum radiatum, CA3r = CA3 stratum radiatum, CA3l = CA3 stratum lucidum, GDmb = Gyrus dentatus mediane blade, GDlb = Gyrus dentatus laterale blade, EC = Entorhinal Cortex

Data on synaptic density are presented in **Fig. 2**. 

## Discussion

The behavioral data, independent of the parameter evaluated, clearly indicated that chronic daily treatment with N-PEP-12 is able to improve learning and long-term memory. The decrease in length of the swimming path and escape latency between the first, the second and the third training course, always with a time difference of one month, also indicated an improved procedural memory. That basically meant that the animals were able to remember the task they had to perform and did not have to re-learn the complete task procedure. It was of great importance to note the accordance between the escape latency and the length of the swimming path, which basically indicated that no bias, by differences in motor function, between the two treatment groups impacted the outcome.

The results obtained in the beginning of the second treatment course can also be interpreted as a combination of improved long-term memory and accelerated acquisition. However, it has to be considered that only one trial was used for the calculation so that training effects for the interpretation of the results can be neglected.

The fact that both groups reached the same improved level of performance in the third training course can be explained by the fact that although all of the animals were aged, they were completely healthy, without any obvious disturbances and that in terms of the MWM performance, a bottom effect was already reached. Due to repeated training and daily handling, all of them improved their cognitive performance. However, different intensity of training was required depending on whether rats received N-PEP-12 or saline treatment. This was most striking in the second training course when chronic ingestion of N-PEP-12 led to a facilitated acquisition of new information and improved memory storage. The behavioral data were perfectly confirmed by the immunohistochemical findings evaluating the synaptic density in the hippocampal formation and entorhinal cortex. In neurophysiology, the relationship between the cognitive performance and brain connectivity was an approved fact. It was impressively demonstrated that in different models of brain aging, the decline in synaptic density goes along with disturbances of memory and learning function.

Despite the disadvantage of training and retrieval overlay effects, the longitudinal trial design revealed that treatment with N-PEP-12 led to a faster task learning when compared to the controls. This improved cognitive performance correlated well with morphological changes, indicating an increased connectivity of neurons, exactly in those areas that are responsible for spatial navigation, for learning and memory formation. It is known that repeated training clearly improves the outcome in behavioral test, but also has an effect on neuronal sprouting and synaptic plasticity. Therefore, it can be concluded that a cross sectional design with only one acquisition/ retrieval course would have shown even more pronounced N-PEP-12 effects than the used trial design. 

The significant increase in synaptic density might have long-term consequences for brain aging, slowing down overall cognitive loss and reducing the risk of cognitive performance decline with age. Increased synaptic plasticity might also improve the ability of adaptation to changed environments for the aged rodents. It is of interest to explore what are the consequences of withdrawal of N-PEP-12 for synaptic connectivity in further studies. How long can the improved brain plasticity be maintained and over which time periods are behavioral changes detectable?

What should be also taken into account is that the treatment started in an advanced age, when already detectable loss of synaptic density and cognitive performance was present as shown in previous experiments. The Long Evans rats started with the cognitive decline already at the age of 12 months. It can be speculated that earlier onset of treatment and/ or prolonged treatment courses would have increased the overall effects of N-PEP-12.

The data of the histological examination were in full accordance with the previously reported findings from tissue culture, where also increased neuronal sprouting was induced by the biologically active peptides of N-PEP-12. This indicated that sufficient amounts of the peptides survived gastrointestinal passage, were absorbed in the intestine, and were transported to the brain. 

Daily treatment with N-PEP-12 was well tolerated and no side effects were detected in any of the animals. 

## Conclusions

Long-term ingestion of growth factor like peptides is well tolerated and is able to provide neurotrophic stimulation to the brain. This improves synaptic plasticity in a significant way, also restores, and enhances cognitive performance as shown by improved learning and memory in the N-PEP-12 treated animals. In summary, N-PEP-12 constitutes a novel supplement that may help maintain memory and learning performance during aging and might reduce the risk of cognitive function loss associated with the aging process.

**Acknowledgments**

Thanks go to Mariella Ribul for her contribution to the histological data of this publication.

## References

[1] Azevedo FA, Carvalho LR, Grinberg LT, Farfel JM, Ferretti RE (2009). Equal numbers of neuronal and nonneuronal cells make the human brain an isometrically scaled-up primate brain. The Journal of comparative neurology.

[2] Pakkenberg B, Pelvig D, Marner L, Bundgaard MJ, Gundersen HJ (2003). Aging and the human neocortex. Experimental gerontology.

[3] Morrison JH, Baxter MG (2012). The aging cortical synapse: hallmarks and implications for cognitive decline. Nature Reviews Neuroscience.

[4] von Bohlen, Halbach O (2010). Involvement of BDNF in age-dependent alterations in the hippocampus. Frontiers in Aging Neuroscience.

[5] Geinisman Y, Berry RW, Disterhoft JF, Power JM, Van der Zee EA (2001). Associative learning elicits the formation of multiple-synapse boutons. The Journal of Neuroscience.

[6] Toni N, Buchs PA, Nikonenko I, Povilaitite P, Parisi L (2001). Remodeling of synaptic membranes after induction of long-term potentiation.

[7] Woolley CS, Wenzel HJ, Schwartzkroin PA (1996). Estradiol increases the frequency of multiple synapse boutons in the hippocampal CA1 region of the adult female rat. The Journal of comparative neurology.

[8] Windisch M, Hutter-Paier B, Grygar E, Doppler E, Moessler H (2005). N-PEP-12 - a novel peptide compound that protects cortical neurons in culture against different age and disease associated lesions. Journal of Neural Transmission.

